# Anterolateral thigh flap to the rescue: anterolateral thigh free flap for secondary reconstruction of facial defect post oncosurgery—a case report

**DOI:** 10.1186/s13256-025-05658-5

**Published:** 2025-12-06

**Authors:** Divya Suvarna Dixit, Firoz Borle, Nitin Bhola, Bhushan Mundada

**Affiliations:** 1 Department of Oral and Maxillofacial Surgery, Sharad Pawar Dental College and Hospital, Datta Meghe Institute of Higher Education and Research, Sawangi, Wardha, India; 2 Department of Plastic Surgery, Jawaharlal Nehru Medical College and Hospital, Datta Meghe Institute of Higher Education and Research, Sawangi, Wardha, India

**Keywords:** Orocutaneous fistula, PMMC flap, Flap dehiscence, Free flap, Anterolateral thigh flap

## Abstract

**Background:**

Orocutaneous fistula is a challenging postoperative complication often encountered in head and neck reconstruction, typically resulting from surgical site dehiscence, infection, or compromised flap vascularity. While the pectoralis major myocutaneous flap has historically been a reliable option for reconstruction, its failure—though uncommon—can lead to persistent fistula formation. This case highlights the effective use of combined local tissue rearrangement and free anterolateral thigh flap for successful secondary reconstruction following pectoralis major myocutaneous flap failure.

**Case presentation:**

A 31-year-old Indian male patient, previously treated with composite resection and pectoralis major myocutaneous flap reconstruction for carcinoma of the buccal mucosa, presented with orocutaneous fistula due to pectoralis major myocutaneous flap dehiscence and partial necrosis. Conservative wound care failed to achieve closure. The patient underwent secondary reconstruction using local tissue rearrangement and a free anterolateral thigh flap. Postoperative recovery was uneventful, with successful closure of the fistula and restoration of orofacial continuity.

**Conclusion:**

This case underscores the importance of timely secondary intervention using free tissue transfer and local flap adjustment in managing orocutaneous fistula following pectoralis major myocutaneous failure. The combined approach provided durable closure and functional restoration, offering a viable option in complex salvage scenarios.

## Introduction

Reconstructive surgery is essential for restoring function, aesthetics, and quality of life in patients with facial defects resulting from trauma, congenital anomalies, or oncological surgeries [1]. Composite defects in the head and neck region following aggressive resections often require the reconstruction of multiple layers, including the intraoral lining, osseous structures such as the mandible or maxilla, and soft tissue or skin coverage [2]. Reconstructive techniques for facial abnormalities must guarantee sufficient coverage while preserving symmetry, texture, and pliability that are similar to those of original tissue [3].

Microvascular free flap reconstruction is an established gold standard for head and neck reconstruction. Free flap reconstruction provides ease of flap inset, freedom to contour the flap to the defect requirements, reliable blood supply, and lesser donor site morbidity. The pectoralis major myocutaneous (PMMC) flap is largely confined to as a salvage option in head and neck reconstruction [4]; however, in resource-constrained settings, PMMC flap is often utilized for head and neck reconstruction. The reasons for such an approach are usually owing to lack of expertise in microvascular reconstruction and cost constraints [5].

The vast surface area and volume requirements of major defects are frequently not adequately addressed by traditional pedicled flaps. These flaps are often morbid and have propensity for wound dehiscence due to the traction on the suture line from the donor site [6]. When utilized in bi-paddle fashion for a large composite defect, there is often a change of suture line dehiscence at the superior aspect of skin defect, leaving a large orocutaneous fistula. Management of this situation is challenging owing to lack of local flap resources, need to provide both intraoral and extraoral lining, inflamed ipsilateral neck, and PMMC muscle covering the neck blood vessels, making access to them tricky.

Here, we report one such case where the orocutaneous fistula (OCF) was managed with a combination of tissue rearrangement and free anterolateral thigh (ALT) flap with anastomosis to opposite neck.

## Case presentation

A 31-year-old Indian male presented with a soft tissue defect on the right side of the face, accompanied by an orocutaneous fistula. He had undergone surgical treatment for carcinoma of the right buccal mucosa 1 month prior, which included resection of the lesion, marginal mandibulectomy, maxillectomy, right-sided neck dissection, and reconstruction using a pectoralis major myocutaneous (PMMC) flap.

At a routine follow-up 10 days postoperatively, wound dehiscence with partial necrosis of the outer paddle of the PMMC flap was noted, resulting in a 7 cm × 6 cm extraoral skin defect. The patient also presented with signs of orocutaneous fistula formation, difficulty in closing his mouth, salivary drooling, and inflamed surrounding tissue. In addition to these functional limitations, the patient expressed dissatisfaction with the aesthetic outcome, prompting consideration of secondary reconstruction.

Given the patient’s young age, the size and complexity of the defect, and his refusal of forehead or scalp flap options owing to concerns about visible scarring, microvascular free flap reconstruction was planned. An anterolateral thigh (ALT) flap was chosen for secondary reconstruction.

Since the initial PMMC flap covered the vessels in the ipsilateral neck, the contralateral (left) side of the neck was explored to identify suitable recipient vessels. The margins of the defect were surgically refreshed, and a turn-over flap was fashioned from the viable portion of the PMMC outer paddle to reconstruct the intraoral lining. Commissuroplasty was simultaneously performed to restore the oral commissure.

**Fig. 1:**
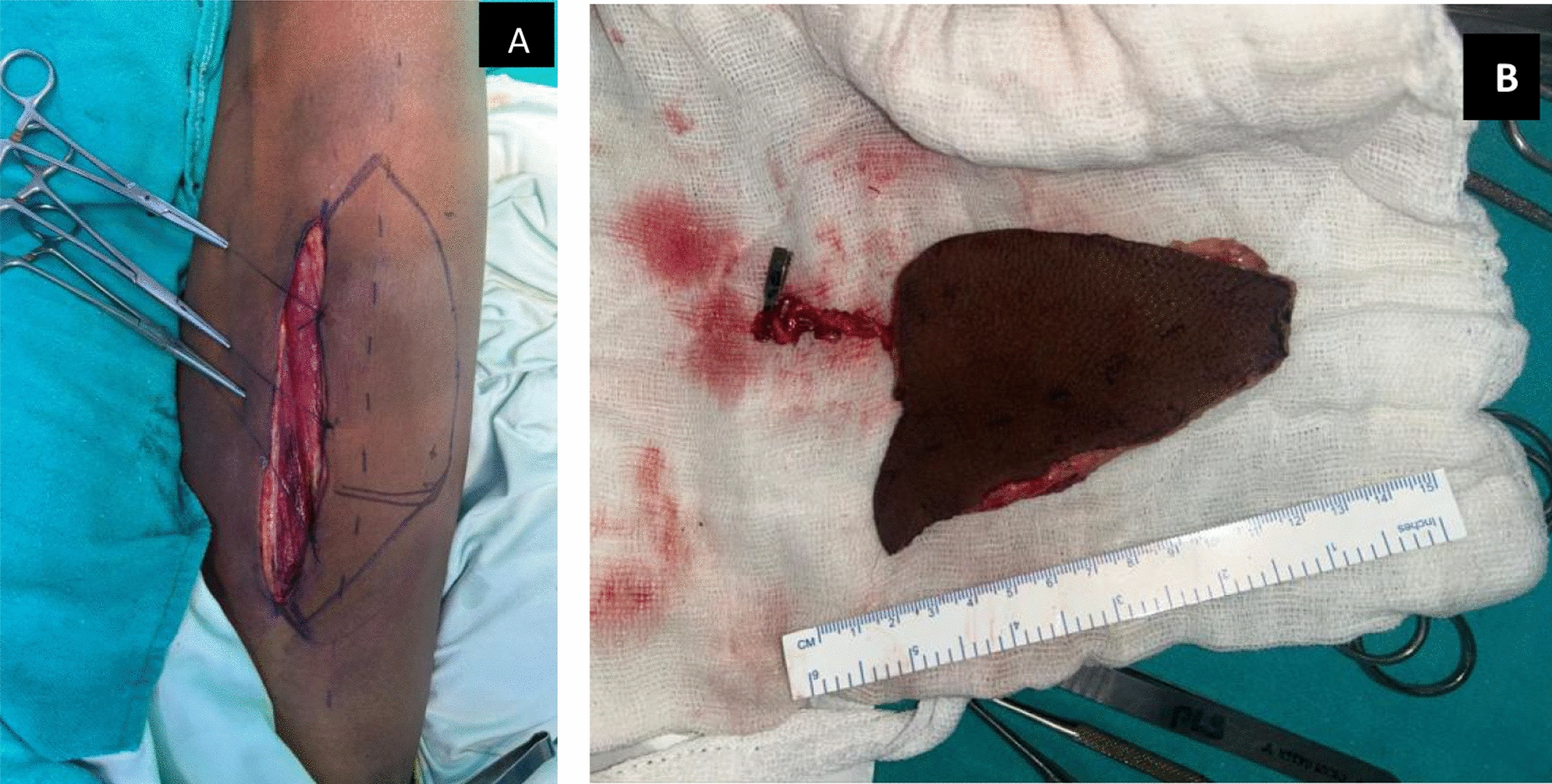
**A** Anterolateral thigh flap harvest. **B** Reconstruction with anterolateral thigh flap

A 10 cm × 6 cm ALT flap based on a single septocutaneous perforator arising from the descending branch of the lateral circumflex femoral artery (LCFA) was harvested. The flap was inset to cover the extraoral defect, and microvascular end-to-end anastomosis was carried out using the left facial artery and vein. The donor site on the thigh was closed primarily (Fig. 1A, B).

The postoperative period was uneventful. On short-term follow-up, the flap demonstrated excellent integration with favorable functional and aesthetic outcomes. Donor site morbidity was minimal. With physiotherapy support, the patient resumed normal oral function and expressed high satisfaction with the result. He subsequently completed adjuvant radiotherapy as per oncological planning.

## Discussion

Microvascular free flaps remain the gold standard for reconstruction in complex head and neck defects, particularly in cases where locoregional flaps may be a viable but suboptimal option [7]. Although locoregional flaps such as the pectoralis major myocutaneous (PMMC) flap are often employed for orocutaneous fistula closure, their use in extensive or recurrent defects is frequently associated with wound dehiscence and persistent fistula formation. Orocutaneous fistulas present unique challenges as they not only compromise function and aesthetics but also create a risk for chronic infections and impede quality of life. Such complications not only compromise the surgical outcome but also result in significant delays in initiating adjuvant therapy, directly impacting overall patient prognosis [8].

In these challenging scenarios, innovative and tailored reconstructive strategies are essential. The skin paddle of the PMMC flap, for instance, can be creatively utilized as a turnover flap to recreate intraoral mucosal lining [9]. The external defect can then be addressed using additional locoregional flaps such as the deltopectoral, scalp, or forehead flaps. However, despite their utility, these flaps are often associated with significant esthetic deformities and typically require multiple staged procedures. This increases patient morbidity, prolongs hospitalization, and further delays the initiation of adjuvant treatment. Moreover, patients may refuse such options owing to the potential for visible disfigurement [10].

Therefore, the primary goal in these cases should be to achieve rapid and effective wound closure that permits early initiation of adjuvant therapy and facilitates timely rehabilitation [11]. Microvascular free tissue transfer fulfills these requirements owing to its reliable vascularity, adaptability to varied defect geometry, and single-stage reconstruction potential. Among the microvascular options, the anterolateral thigh (ALT) flap has emerged as a workhorse owing to its consistent vascular anatomy, which offers several advantages that make it an excellent choice for reconstructive surgery, reliable vascularity, large cutaneous territory, and the unique anatomy of the thigh, which allows for multiple harvesting techniques [12]. The choice of tissue components—cutaneous, subcutaneous, or muscular—can be customized to meet the specific requirements of the defect. This adaptability, combined with other key features, has made the ALT flap a preferred option for addressing complex reconstructions, including orocutaneous fistulas [13].

A common technical challenge in salvage reconstructions is the availability of suitable recipient vessels. The ipsilateral neck often presents with dense fibrosis or inflammation or may be obscured by remnants of previously used flaps such as the PMMC, complicating vessel dissection. In such cases, accessing contralateral neck vessels is often the most straightforward and safest approach to secure reliable microvascular anastomosis [14].

## Conclusion

ALT free flap reconstruction provided reliable closure of a large orocutaneous fistula in this case of secondary head and neck reconstruction following PMMC flap failure. The flap offered excellent functional restoration, including improved oral competence and speech, along with aesthetically acceptable contour and symmetry. Minimal donor site morbidity and a smooth postoperative course further highlighted its clinical effectiveness. This outcome reinforces the ALT flap’s value as a versatile and dependable option for complex secondary reconstructions in the head and neck region, particularly when both intraoral and extraoral components are involved.

## Data Availability

The data utilized in the development of this manuscript are available upon request from the corresponding author.
